# The importance of relationships in mental health care: A qualitative study of service users' experiences of psychiatric hospital admission in the UK

**DOI:** 10.1186/1472-6963-8-92

**Published:** 2008-04-25

**Authors:** Helen Gilburt, Diana Rose, Mike Slade

**Affiliations:** 1 Health Service and Population Research Department, Institute of Psychiatry, Kings College London, London, SE5 8AF, UK

## Abstract

**Background:**

While a number of studies have looked at life on service users' experiences of life on psychiatric wards, no research exists that have approached these experiences from the user perspective since the introduction of community care.

**Methods:**

This user-led study uses a participatory approach to develop an understanding of the processes and themes which define the user experience of hospitalisation. Nineteen service users who had all had inpatient stays in psychiatric hospitals in London were interviewed in the community.

**Results:**

Relationships formed the core of service users' experiences. Three further codes, treatment, freedom and environment defined the role of hospital and its physical aspects. Themes of communication, safety, trust, coercion, and cultural competency contributed to the concept of relationships.

**Conclusion:**

Relationships with an individual which comprised effective communication, cultural sensitivity, and the absence of coercion resulted in that person being attributed with a sense of trust. This resulted in the patient experiencing the hospital as a place of safety in terms of risk from other patients and staff. Barriers to positive relationships included ineffective and negative communication, a lack of trust, a lack of safety in terms of staff as ineffective in preventing violence, and as perpetrators themselves, and the use of coercion by staff. This unique perspective both acts as a source of triangulation with previous studies and highlights the importance of the therapeutic relationship in providing a safe and therapeutic milieu for the treatment of people with acute mental health problems.

## Background

The patient experience is increasingly being recognised as an important factor in developing and providing excellence in healthcare. In the UK, improving the patient experience is declared to be central to everything the NHS does and the newly formed 'Improving the Patient Experience' initiative, a collaborative project across the whole National Health Service, is recognition of this [1].

A review of studies focusing on what is known about psychiatric in-patient care revealed five methodological approaches to investigating the experiences of patients in acute admission wards [2]. These included participant observation, discourse/conversation analysis, in-depth or semi-structured interviews with patients, quantitative surveys of patients, and 'snapshot' observational methods. Surveys have sought patient ratings on broad sets of topics including general satisfaction, physical environment, staff-patient relationships, information, treatment, restrictiveness, autonomy, individualisation, and control and compulsory admission [3-6]. In addition, qualitative interviews have largely focused on making sense of specific themes such as violence, involuntary treatment, coercion, care, admission, treatment, restraint, seclusion, observation, mixed sex units, environment and rules, daily life and relationships with staff. However a number of authors have identified difficulties with the current knowledge of the patient experience of inpatient psychiatric services. The variables on which survey measures are based have been criticised for their lack of theoretical underpinning [7], and both these variables and the themes identified for qualitative investigation are largely clinician-derived [8]. Divisions in the views of patients and professionals in terms of what variables and themes are important mean that the resulting studies may be a poor representation of the user perspective [9,10]. Quirk and Lelliot [2] also concluded that, of the research they reviewed, the emphasis was very much on clinical practice and the worlds of professionals and that there was a real need for in-depth interview studies involving patients to understand the meaning of in-patient care to these people.

Participatory research aims to bring into the foreground the lived experience and indigenous knowledge of participants. Participatory approaches invite the participation of stakeholders in research and in doing so act to recognise the role of power in the research process and challenge this. In emancipatory research, participants control all aspects of the research process as opposed to simply participating. The roots of emancipatory research lie in feminist theory [11]. Like that of feminist, black, and other established new paradigm research fields, emancipatory research maintains that a different view may be obtained when the researched become the researchers. Emancipatory research in the mental health field has taken the form of user-led research. The benefits of user-led research are that the work done is relevant to the concerns of service users [12], the reciprocal relationship between interviewer and interviewee puts both on a more equal footing [13], aids the ability to achieve real empathy [14] and can elicit the collection of data not otherwise accessible [12]. During data analysis the researchers background sensitises them to themes and concepts that may otherwise not be apparent. Finally, revisiting the subject of patients' experiences from the unique perspective of a user-led study serves not just as a source of new data but also allows triangulation with previous research.

The aim of this paper is to explore the experiences of admission to acute psychiatric hospital from the perspective of services users. Key themes are identified and triangulated with previous research to confirm and expand our current understanding of the impact and consequences of mental health care in psychiatric hospital.

## Method

### Design

As a user-led study a participatory research approach was used. Both HG and DR are service users with experience of admission to psychiatric hospitals. Both researchers were involved in the design and analysis of the research study. The interviews were undertaken by HG. Particular notice was taken of the power imbalances involved in the research process and efforts were made wherever possible to empower participants. The data was analysed using thematic analysis. One of the benefits of thematic analysis is its flexibility and independence of theory and epistemology which can potentially provide a rich and detailed, yet complex account of data. The unique user-focus of this study resulted in the use of an inductive approach to the identification of themes where the themes are strongly linked with the data themselves. Consequently the relevant literature is only reviewed after the analysis is completed [15].

### Participants

A total of 19 service users who had each experienced admission to a psychiatric hospital in England were recruited. Glaser [16] suggests that in the initial stages of a study, researchers should begin by talking to the most knowledgeable people to get a line on relevancies and immerse oneself in a rich supply of data. Participants were identified through volunteer sampling because there were no data to direct what further information should be sought and explored. Two sampling strategies were used. Mental health resource centres were identified as suitable recruitment sites as Catty and Burns [17] reported that many clients of such services had used, and continued to use, inpatient services. In addition an advert was placed in a local mental health charity newsletter to reach a larger audience including mental health service users who were working and unlikely to be using mental health resource centres. The advert described the study and the researchers contact number for more information. This study formed part of a larger study into residential alternatives to traditional psychiatric hospitals and as a consequence, resource centres and participants were contacted in the London boroughs of Haringey, Croydon, Havering, Camden, Islington and Harrow in which residential alternative services were also located. Following initial contact with each resource centre, the researcher attended a community meeting at each of the services to introduce the study to service users and answer any questions. The researcher returned to each service on an arranged date to conduct the interviews. Prior to each interview, each participant was given an information sheet and their written consent was sought. Seventeen participants were recruited from four mental health resource centres and a further two participants through the newsletter.

Ten of the participants were men and nine were women; 13 participants were white British, one was white European, three were Black British and two were Asian British. The majority were between the ages of 25 and 60, while three were above the age of 60. All but two adults of working age were unemployed. Collectively participants had had inpatient stays in over 10 different hospitals in England and comprised both service users who had had a number of admissions over several years, and those who had more recently come into contact with inpatient services for the first time.

### Data collection

We initially conducted a focus group of ten people at a mental health resource centre. A further nine participants took part in face-to-face interviews. While participatory approaches focus on the role of power dynamics in the research process, Hoffman [18] argues that during interviews both interviewers and interviewees wield these powers at different times and in various ways. As such the process of reflexivity becomes crucial for the interviewer to recognise the power dynamics through the research process and work towards the empowerment of participants. As an academic researcher and initiator of the contact, the interviewer held a certain amount of power. A number of strategies were employed in order to help move the researcher and participants to a more equal sharing of power. Interviews were scheduled at a time and location of the participant's choice and an open and unstructured interview format was used. Each interview opened with the request "Tell me about your experiences of being an inpatient," allowing participants to determine the direction of the conversation. The researcher assumed an open stance towards the participants as well as sharing personal details and answering questions both during the interview and afterwards. At the conclusion of each interview participants were paid a nominal sum for their time and contribution.

After each interview the researcher wrote memos of personal reflections and theoretical insights. Data collection and analysis were iterative with each step of analysis. In each subsequent interview, the researchers' understanding of the themes arising through coding and reflection was shared with participants to seek further understanding of the themes for explanation in later interviews.

### Analysis

All interviews were audio-taped and transcribed verbatim by the first author helping familiarise the researcher with the data. Responses were analysed using the inductive thematic analysis procedure described by Hayes [19]. First, the data was read carefully to identify meaningful units of text relevant to the research topic. Second, units of text detailing the same issue were grouped together in analytic categories and given provisional definitions. The same unit of text could be included in more than one category. Third, the data were systematically reviewed to ensure that a name, definition, and exhaustive set of data to support each category were identified. The inductive thematic analysis resulted in 27 categories, which were grouped into 8 key themes.

During the entire coding process, memo writing was used both as an analytical tool to record concepts, themes and more abstract thinking about the data, but was also used as a means of reflection, to record the researcher's own beliefs and experiences. Both the data and memos were shared with authors' research supervisor DR and discussed until consensus was reached.

## Results

One of the most important findings of the current study was that when participants talked about their experiences of hospital, they did so largely within the context of the people that they had encountered during their admission. Five out of the eight themes related to relationships, these included communication, coercion, safety, trust, and culture and race. One theme, treatment, highlighted the role of admission to hospital. Two further themes are structural, providing an understanding of the environment of hospital and include the themes, environment and freedom.

### The role of communication

Communication was highlighted by all participants and constituted the greatest number of coded sections, constituting a third of all coded sections. This illustrates its importance to service users. Communication comprised three specific activities, listening, talking and understanding. In order for communication to take place the participants must be approachable and/or initiate contact.

"As soon as you come they can see that you are angry. Then someone will say, sit down, let's talk about it, make a cup of tea."

Obstacles to communication included unavailability.

"The staff work hard at trying to stay away from the clients was my opinion. Be in their office as much as they could."

Listening was rated highly by service users. The ability to listen was described as a characteristic of being human and service users who had the experience of being listened to described feeling respected. Conversely, one service user rated a whole service negatively because he felt the staff did not listen to him. Listeners who were open, non-judgemental and not patronising were valued.

"...they have their own agenda about what I ought to do and the way I ought to be, rather than let me talk about my problems. I need someone to listen to me and I can't get them to listen to me."

"I took away from that was the feeling, the humanness of everybody, the commitment of everybody and I was just so moved by the willingness of so many people to sit and listen to whatever it is however horrific it might be or however banal it might be they were willing to listen to it all and not patronise me."

The process of talking was by far the most prominent aspect of communication and represented over half of the codes linked to communication. It was identified as important by all participants. Talking was described as therapeutic, but only if the service user was listened to and understood. Service users who were understood valued relevant advice and information.

Interviewer: *"What did you like about the staff?"*

Service user: *"The actual nurses, I think they sort of, understood"*

Thirteen people identified numerous instances of a lack of, or poor communication, between service users and staff. In contrast there were no such negative references to communication between service users. One of the key factors in being able to communicate with other users was the shared experience.

"And when I was there I met patients that I could sort of talk about things between us and she'd know what I meant."

There is an overlap between the topics communication and coercion. Positive experiences of communication led to a person feeling supported and cared for, however, coercive communication, such as the use of threats, was experienced entirely negatively.

### Coercion

Coercive experiences were reported by all of the service users interviewed. Objective coercion, such as involuntary commitment and treatment, was often negatively reported, but the coercion of being detained was not attributed to the legal process involved but rather to coercive events that service users were subject to as a consequence of detention. Such events included restriction of freedom and compulsory treatment.

"See the first time I ever went in there I think I was on a section actually and it felt horrible. It felt horrible because I was locked away for so many days and I couldn't go out and be free."

Four men and one woman described being restrained. All were involuntarily detained when the restraint occurred. All counts of restraint were accompanied with forcible medication. Restraint was described as a form of assault and in one case as leading to physical injury.

"I wasn't restrained, I was attacked."

"They wanted to tear me to pieces and I have arthritis of the shoulder to prove it."

Perceived coercion was reported by both compulsorily and voluntarily admitted patients. It followed the form of threats of non-physical force or of consequences resulting from disobeying staff wishes. Perceived coercion was described by service users as being "hypnotised" and "brainwashed" and reactions to perceived coercion were referred to by two people as "playing the game". The most common threat experienced by voluntary patients was of being detained. This was reportedly used to coerce patients into hospital on a voluntary basis, and once admitted into remaining in hospital or receiving unwanted treatment.

"First of all I didn't want to go in and my GP at the time said either you go in or I section you."

"...and my psychiatrist said if you don't take your tablets I will section you and give you ECT under section."

The use of non-physical force also represents a form of perceived coercion.

"I was forced to take medication that was causing me a lot of discomfort."

A final category of coercive experiences includes reports in which a clear abuse of power and trust is taking place with little justification. Three people report such incidences. One incident described a staff decision not to treat a patient in considerable distress.

"There was a nurse who I witnessed saying to him, while you were in this catatonic state and before you got ECT you weren't aware of this but one of the other nurses pulled you off the ground and was taunting you, pulled you off the seat and was taunting you, breaking up your cigarettes in front of you to try and get you to react."

There was a link between the codes for "coercion" and "safety". Descriptions of perceived coercion that were associated with feelings of a lack of safety, rather than actual coercive practices such as restraint.

"Knowing that if I tried to leave I would just get sectioned. So it was a terrifying place, position to be in."

### Safety from self, safety from others

All participants talked about safety. An expectation of hospital was that it would be safe, with service users seeking safety both from themselves and from staff or other patients.

"So I felt, you know, being in hospital was one way of keeping me safe."

Safety in hospital was always spoken about with reference to other people. Social contact could instil a sense of safety in some people, but in others contributed to a lack of safety, and its perception depended on the nature of the contact.

"I need people around me so I don't go and stab myself or do anything really stupid."

"The first thing I got when I was up there was threatened by a bloke."

A lack of safety was associated with ward-based violence, and the feeling of fear. Four participants reported acts of violence and aggression perpetrated by themselves towards members of staff and inpatients, while another four describe incidences of being subjected, or witnessing other patients being subjected, to violence by both staff and other patients. Experiences of violence were always accompanied with a feeling of fear. All but three people described feeling fearful while in hospital. Fear was described as a contributing factor to perpetrating violent acts and as a consequence of experiencing violence.

"I was very frightened and that made me verbally aggressive."

"I was safe in hospital until somebody, some other patient tried to strangle me."

A lack of safety and the experience of fear led to aggression and to one person absconding from hospital.

"Um, scary. Couldn't wait to break out and just disappear like. I even found myself escaping the hospital one night and was crossing the M25 believe it or not."

Both men and women described the feeling of being vulnerable on wards where there was a predominance of men.

"It felt positively threatening to be in a mainly male environment with little support and understanding of how vulnerable, in reality, you were in that situation."

Men described being attacked by other men. However, the one woman who describes being attacked the victim of a fellow inpatient on an all female ward.

Fear was associated with a feeling of not being in control. In addition to situations in which there was a risk of violence, fear of staff was reported when they provided unpleasant medication, and treated people coercively.

"I felt frightened of the doctors, they were putting me on drugs that had terrible reactions. I felt frightened."

While a lack of control elicited a feeling of fear, if that situation was contained and controlled by someone else, the fear could be managed and was deemed acceptable.

"It was very scary so you did need that containing place if you are going to be challenged to that extent. It was terrifying."

In addition to service users' experiences of lack of safety and the associated fear, three participants reported observed fear of patients among staff. Once again this fear is attributed to a perceived lack of control by staff and included fears of patients harming themselves and others. Staffs' fear of patients' behaviour resulted in their use of coercive measures.

"And I think a lot of the fear is from a lot of the consultants is that if somebody does kill themselves they are accountable, they haven't done their job to what is written down to their job."

"I think they were scared when I was unpredictable at the time. So I got harsh treatment."

Interviewer: *"Do you think that in hospital the staff were frightened of you sometimes?"*

Service user: *"No I don't think so because they know they can get a man with an injection and just knock me out."*

### Trust

The word trust was used by five service users in their narrative but instances of trust and mistrust of others in hospital were identified by all participants. Trust was described as important in providing a positive experience and mistrust contributed to a negative experience of being an inpatient. Service users' attributions of trust or mistrust were described only in relation to staff.

"Now she leant me money, I paid her back of course, and she trusted me."

"Trust" was linked with the codes "safety" and "coercion." Two service users describe situations in which they felt their safety was at risk. In one account staff were seen as able to contain and deal with the situation and were attributed with a sense of trust. In the other account the staff were seen as allowing the situation to escalate and were mistrusted.

"There was a lot of tension in the air, a lot of fear, most of the patients tried to disappear off to their rooms or out of the way because this guy was really going to blow and everyone knew it. I decided it wouldn't matter where I was on the ward, there was nowhere for me to lock myself in, the nursing staff didn't give a damn so the only option open to me was to run away, which is what I did."

Staff that were trusted by service users were described as being professional, able to manage situations in which the safety of patients was at risk, flexible, non-coercive, committed, and caring about patients. The use of coercion by staff led to a sense of mistrust.

"It wasn't until I agreed to see my consultant that they allowed me out of the hospital at all. That was a sanction to force me to see my consultant that I don't wish to work with. I distrust my psychiatrist that much."

Although trust was not overtly attributed to other patients, it is clear that in many cases there was an atmosphere of trust between patients that was valued.

"This guy, lovely guy who had taken a major overdose had wet the bed and he'd come to me to say that he was so upset about having done this and couldn't approach the nursing staff because of their attitude."

### Treatment

All but one of the participants recognised that the purpose of hospital in part was to provide treatment. However, trust in staff to treat patients appropriately was not always apparent.

"Because of the people in there were more ill than I was. You can see the nurse don't know what to do for them."

Treatment was composed of two subcategories "medication" and "therapies." Medication was a central part of the experience of being an inpatient and seventeen participants spoke about their experiences with receiving medication while in hospital. There was general acceptance of medication in the treatment of mental illness.

"I suppose in the end what made me well was the tablet form, the medication."

However, there was also dissatisfaction expressed about the types of treatment received and the process of receiving treatment. Six participants described potential overmedication leading to feelings of being "doped up."

"I wasn't able to do anything, only take the tablets and be like a zombie all the time."

There was a strong link between the codes for medication and communication. The value of effective communication in discussions about medication is highlighted by two patients.

Service user: *"You know they wanted to put me on olanzapine, or the other antipsychotic thing, and I didn't want that. Because I've had it before and it was absolutely awful. It's the worst drug I've ever taken. And I didn't want to go there so I refused all that kind of, any medication or tablets."*

Interviewer: *"And they respected that?"*

Service user: *"Yes they respected that. Which was good."*

"I assaulted a patient, which got me on a section. ...What did the consultant do but put me on Depixol and it had a horrific effect on me, absolutely horrific. I can't blame everything on the medication, I know it was wrong of me, and they put me on it against my will, my mothers."

Effective communication is also of prime concern in capacity to consent to treatment, and specifically to receiving ECT. The following participant describes being asked to sign a consent form to receive ECT while actually not having capacity

"When I'm not well, then I'm out of it, I'm not here. Like when I was in one example, when I was in ******, the psychiatrist got me to sign a form to say I'll have ECT. My brother said to him, you could get her life away at the moment, but he had to have me sign it."

There was a strong link between medication and coercion. All physical restraints reported were followed by forcible injection and several people reported perceived coercion in receiving treatment.

In addition to treatment with medication or ECT four people also highlighted a need for talking therapies while in hospital.

"As I started, I mean there were some very ill people in St ***'s and I wasn't offered any kind of counselling or psychotherapy."

Therapies that were spoken of positively were founded on good relationships with the facilitator. This included a group based on the 12-step model run by a nurse who had been a service user herself. Therapies spoken of disparagingly included art, and music therapy. While the art therapy was not in itself ridiculed, it was deemed worthless as an activity by the following participant due to lack of communication and understanding by staff

"So at art therapy, so I was drawing a picture of the crucifixions. You know like, there's nothing wrong with that it's Easter it's got to be accepted by everyone. And they said to me, why are you drawing that? So I said, its Jesus, remember it's Jesus when he died. You know I didn't go round the trees. He said, but this picture, are you feeling like at death's door, are you feeling like you are crucified or something. I said, no I'm just drawing because of Jesus my hero dying at the cross. But they wouldn't have that, they tried to look into, thinking I was crucified inside. And I got so fed up with them and things."

### Cultural competency

Six participants raised issues associated with cultural competency in hospital and all of these experiences were negative. Experiences described include a lack of understanding by staff, and racism. A lack of cultural awareness and sensitivity by staff is demonstrated in the narrative of a young Black African woman describing the difficulties she faces as a result of her belief that her mental illness results from possession and the use of voodoo.

"It was like a misunderstanding, they didn't want to believe that the unknown, the unknown, meaning someone who like deals with like magic or things like voodoo, that's what sort of like brought this all about."

Two service users remarked on the difficulties faced in being nursed by non-British staff and this was explained by one interviewee as due to differences in cultural beliefs about the origin of mental illness. Finally, racism towards ethnic minority patients was reported as an experience by ethnic interviewees and witnessed by white interviewees.

"You know you've got to be conscious of being black, you've got to be victimised for being black, and therefore we'll hurt you, intended to feel like this, because it's a kind of racism. ...And that's what I experienced in the psychiatric system."

"There were a lot of black men in the system at the time, again they were treated worse than I was because I was a white woman."

### Freedom

Twelve participants spoke about freedom while in hospital. The focus was primarily on physical freedom, the freedom to be outside, or to leave the unit. Such freedoms were viewed both as a basic human right, and also therapeutic in reducing feelings of confinement and being in touch with the environment. Conversely, a lack of freedom could induce mental distress.

"I enjoyed the fact that we were allowed a certain amount of freedom as in we could say to the staff person in charge, could say, oh I'm popping down the shops for 5 minutes, then they'd just let you out."

"When I was first there I was distraught and what really distraughted me was when I weren't allowed to go outside and get a drink or anything like that."

A lack of physical freedom was not expressed only by service users who were compulsorily detained. The environment, staff decision-making and resources contributed to perceived freedom. Some hospitals had no outside space for patients, while other patients, even those admitted voluntarily, were not allowed out. Finally one patient describes being granted escorted leave but being unable to go outside due to the lack of an available staff escort.

"I wasn't allowed outside. I didn't even have an exercise yard."

"If you're a voluntary patient there you are not allowed out."

"I couldn't go out for a walk because there wasn't a nurse available."

Hospitals with a lack of freedom were likened by five people to prisons, with service users fulfilling the role of prisoners receiving punishment.

"It felt like a prison."

"I felt like I had done something wrong, that I was a criminal."

Freedom was concurrent with the codes coercion and trust. A denial of physical freedom was often perceived as coercive, and the denial of freedoms was attributed to a lack of trust in patients by staff.

Interviewer: "And did you find the freedom helpful? I understand at ********* you were more free to do things, how did you find that?"

Service user: *"Yeh, because then they either trust you or they don't."*

### Environment

This category embodies the physical elements of the psychiatric hospitals experienced. The category was a minor one and while it was raised by 10 people, those sections coded were the shortest. With the exception of one report, the environment was only raised as a factor in service users' experience if it was quite poor. Descriptions of the hospital environment included a lack of basic hygiene, old buildings in poor physical condition, overcrowding with a lack of staff, and lack in basic home comforts.

"It isn't nice, it's an absolute disgrace. There are no curtains, in the corridor or the smoking room. The windows are filthy; the furniture's filthy and burnt. It's an absolute dive. It's disgusting and I wouldn't put a pig there let alone a human being."

The effect such surroundings had on patients is clearly expressed by one patient:

"I felt quite low about myself and the surroundings at ****'s are very low and so I felt that I fitted in at first."

## Discussion

While Quirk and Lelliot [2] noted that of the majority of the research on service users' experiences of hospital was negative, the participants interviewed for this study identify both negative and positive experiences of being an inpatient in a psychiatric hospital. Central to the narratives of all interviewees were eight main themes: contrary to previous research on patients' experiences, the themes that predominated related to the emotional not physical environment in which they stayed.

The difference in emphasis in the findings of this study may be due to a number of factors, of which the user-led nature of the research and the use of a user interviewer are important factors to consider. Interviewer influence has been a neglected effect in psychiatric research [20]. Interviewers may influence the type of people who consent to take part, the quality and quantity of interview data. Research suggests that user interviewers may help to engage other users whose voices are not normally heard such as those who feel alienated as a result of their experiences of hospital, and those who would not wish to share their experiences with professionals [21]. During the interview, interviewer variance in terms of race, sex and age can making a difference to the content of the completed interview [22,23] and the experience and enthusiasm of the researcher can influence the length and nature of the interview as well as disclosure by interviewees [20]. An acknowledgement of power differentials between interviewer and interviewee throughout the interview process [18] and an effort to empower interviewees through an emancipatory approach can also affect the traditional relationship between researcher and researched and consequently the narratives elicited [14]. Finally, analysis by insider or outsider researchers, that is, researchers with different standpoints, such as clinical or user researchers can also affect the interpretation of the interview's content and the presentation of results [14]. Confounding factors which ultimately impact on the findings of research studies are a consequence of all qualitative approaches. Such influences are often referred to as limitations of a study, however within emancipatory research the influence of an insider researcher is seen as a strength of the approach. When undertaken with rigor and reported in an open and transparent manner, emancipatory research promotes an understanding of an area from a unique perspective.

The physical and emotional components of patient experiences have only recently being recognised. Initiatives within the NHS aimed at improving the patient experience in hospital have focused largely on the physical environment. However, more recently consultation with patients, public and NHS staff has worked to define the emotional aspects of positive patient experiences and include the need to feel cared for, safe, confident and in control, being communicated with as an equal, and being treated with honesty, dignity and respect [24]. Many of these emotional experiences come as a result of positive aspects of relationships, and this emphasis on relationships in shaping experiences is clearly described by the participants in this study.

The overarching theme of these interviews was that of interpersonal relationships. Human relationships can be argued to be the primary motivational force in life [25]. It is not surprising therefore that relationships while in hospital play an important role in shaping patient experiences. Service users' descriptions of their experiences were largely centred on their relationships with staff or other patients. The importance of relationships cannot be underestimated, with increasing evidence that building and maintaining a strong therapeutic relationship can be an agent for change in itself and leads to positive client and treatment outcomes [26,27]. While each of the themes relating to relationships, depending of the quality of interaction, could affect the relationship in a positive or negative way, coercion was always experienced negatively and had a negative impact on relationships.

Communication was the theme most central to the perception of relationships and an essential ingredient of the patient experience. How a relationship was experienced related to the nature and quality of the communication. Leach [26] recognises the impact that a clinician's behaviour and communication style can have on practitioner-client relationships. He discusses aspects of staff engagement that elicit good communication, trust and rapport with patients, many of which can be said to be of importance to the service users interviewed. Both studies highlight the importance of staff being approachable, non-judgemental, engaging, empathic, respectful of clients' wishes and needs, and the formation of a collaborative relationship. The largely positive relationships of service users in this study with other patients, and staff who had personal experience of mental illness may be indicative of the value of collaboration, self-disclosure by both parties in developing relationships [28].

Both safety and trust were important in influencing the patient experience and the consequences of positive therapeutic relationships in hospital. The issue of safety was key to how relationships were experienced in hospital. With one of the functions of hospital being that of a place of safety, service users defined safety both in terms of safety from themselves and safety from others. The need for social input and the link between social isolation and suicide highlights this importance of safety for those at risk of harm [29]. However, much of the discussion with service users centred on a lack of safety on wards. The role of violence within the mental health system is a largely under researched subject [30]. With the adoption of zero tolerance policies across hospital departments, much of the focus has been on patient perpetrated violence. Although this remains a concern of patients, in this study it was clear from these interviews, and those of Kumar *et al*. [30], that patients are not the only perpetrators of violence. Service users described restraint techniques as a violent act perpetrated by staff towards patients, and service users described resulting injuries. The practice of restraint is under close scrutiny in the UK following a number of high profile deaths e.g. David Bennett case [31]. While the use of restraint may be a necessary force in controlling violent outbursts on wards, service users described no other techniques being used in the lead up to restraint to dissipate the situation. Furthermore, for some service users, staff were instrumental in provoking situations that made violence more likely. Participants described staff winding them up and playing games, a practice that was also found in a study on violence and abuse against social workers [32]. Essex [33] warns that the words and manner of staff can prevent or induce aggression or confrontation.

A further indication of a lack of safety on acute wards was the widespread report of fear by service users. Both women and men felt particularly vulnerable on male dominated wards. The vulnerability of women on mixed sex acute wards has been previously reported [34] and single sex wards are now government policy [35], but the vulnerability of men on male dominated wards is an area as yet unexplored. It is argued that single sex wards may reduce fear and risk of assault for some women but can result in non-therapeutic environments for male patients, and are argued by some researchers to be an inadequate solution to what women really want from mental health services [36,37]. Despite reports of fear, it did not always lead to a negative experience of hospital. The role of staff in maintaining a sense of safety for patients was stressed. An experience of safety was maintained, despite fearful situations arising, when staff demonstrated professionalism in their job and were able to control and contain situations preventing them escalating and affecting other patients.

Another consequence of a positive therapeutic relationship was the experience of trust. Trust can exist between individuals and in a system or institution [38]. The trust spoken about by participants was solely interpersonal trust as a result of relationships in hospital. Trust is the basis of positive social interaction and is necessary for daily life [39], it has even been argued that the practice of medicine is impossible without the trust of patients [40]. For the doctor-patient relationship, trust affects willingness to seek care, reveal sensitive information, submit to treatment, and follow physician's recommendations, and may also affect behaviours and outcomes [41,42]. In common with conceptualisations of trust in staff-patient relationships [43,44], participants expressed the value of being able to talk through experiences with staff and ask questions, involvement in decision making, and having a sense of emotional equality. Participants expressed a marked disparity in the assignment of trust to different people in hospital. Trust was frequently attributed to other service users and although intimated, mistrust of other patients was never actually articulated. In contrast, there was an emphasis on reported mistrust of staff, and even when a patient put the well-being of other patients on the ward at risk, the staff were assigned responsibility for their ability to contain or bring the situation under control. With risk and trust being closely related [45], it is an unexpected finding that service users who present risk on wards, are not identified as untrustworthy. This may however be indicative of the greater risk that staff are perceived to present to patients. Interviewees give a further reason for the attribution of trust to staff in the description of trusted staff as professionals. Trust as an expectation about the future behaviour of a person [46], may be expected of staff as professionals about whom there is a level of expectation. Staff that fail to fulfil their patients' expectations of professionalism may be deemed untrustworthy.

The yearly census of mental health services for inpatients undertaken by the Healthcare Commission has backed up research studies in highlighting higher rates of admission and detention for patients from Black groups with higher levels of seclusion once detained [47]. Service users interviewed here describe discrimination in mental health services attributed to racism, but also the more subtle yet discriminatory impact of a lack of cultural awareness. Like white interviewees, the experience of black and ethnic minority interviewees was also largely defined by the relationships they had experienced in hospital. Cultural awareness is seen as an important factor in developing therapeutic relationships, and argued by Hardy and Laszloffy [48] to be crucial. Since culture is a lens through which a person views the world, it plays a critical role in mental health [49]. Culture defines what is normal and abnormal, the causes of problems, and the appropriate ways to help a person who is disturbed. Service users described a lack of cultural awareness by staff resulting in their expressions of illness, and perception of aetiology, being ignored or misinterpreted. However, it was service users from white backgrounds who highlighted racism towards ethnic minorities within psychiatric hospitals. Such attitudes and practices are argued to be a result of both institutional racism in the NHS [31,50] but may also be inherent in the procedures, practice, and policy governing service delivery [51,52]. For people from black and minority ethnic groups, then, their social identity may be used to prevent more positive therapeutic relations being formed between themselves and mental health professionals.

Despite the emphasis on the role of the therapeutic relationship in patients' experiences, the role of medication and treatment was also recognised. Participants were accepting of medication as an important, if not vital, component in the treatment of mental illness. However, its potentially coercive overuse and use without consent led to negative experiences. Participants stress the importance of communication in continuing consultation with both service user and family.

Participants identified that the main barrier to the formation of a therapeutic relationship was the experience of coercion. Relationships that were perceived as coercive were always described as negative and resulted in negative patient experiences. Coercion comprises objective coercion and perceived coercion. Objective coercion implies the deprivation of liberty, the use of seclusion, restraint and forced medication. Perceived or subjective coercion refers to the patient's experience of being coerced. The inherently coercive nature of the English Mental Health Act [53], makes coercion particularly relevant to detained patients. Reports of restriction of freedom and compulsion to receive treatment by participants who had been detained confirm this association between compulsory admission and experience of coercion reported by other authors [54,55]. In the present study, objectively coercive experiences, such as a reduction in freedom as direct result of detention, were not attributed to the legislation itself but to the purveyors of the coercion. Furthermore, enactment of the legislation in which a sense of safety was instilled by the staff performing the sectioning and experienced in a positive manner by the service user suggests that coercion is not necessarily a function of the Mental Health Act, but of the relationship with the staff enforcing aspects of it. The use of coercion in relationships between staff and service users is not limited to detained patients [53,54]. Both participants who had been detained and those staying voluntarily reported experiences of perceived coercion. In this study, threats of force were the most widely reported form of subjective coercion. Threats have been shown to be positively associated with patients' perceptions of coercion [55]. Subjective coercion was most commonly experienced by voluntary patients when they were threatened with sectioning. Szasz [56] refers to this practice when characterising voluntary hospitalisation as "an acknowledged practice of medical fraud" in part because "a person is forced to sign in... as a voluntary patient under the threat of commitment". In this study participants saw subjective coercion as an inherent, but unacceptable, part of being an inpatient. Their survival under such conditions is described as 'following the rules' and 'playing the game'.

Coercive practices were used both to maintain control of patients, for instance through the use of restraint and seclusion, but also resulted from staff being in a position of power. Inherent in the staff role of maintaining control is a level of power over patients. It is this power imbalance in institutions that leads to abuses and unethical use of coercion [57]. Service users describe several instances in which staff misuse their power to hurt or humiliate patients. The Mind Ward Watch survey [58] reported that 31% of harassment or assault episodes on psychiatric wards were perpetrated by staff. Such temptations and abuses are documented throughout the care industry [59].

Freedom and environment represent the only two physical themes that were identified as affecting the patient experience. Unhygienic environments with a lack of outside space contributed to a perception of hospitals as prisons. More recently, Bowers et al. [60] reported that the sometimes prison like conditions of locked wards can lead to increased rates of self harm and attempted suicide. The deleterious effects of the hospital environment on psychiatric patients was identified nearly 30 years ago by Goffman [61]. Increasing attention is being paid in the design and building of new psychiatric hospitals to the role of the environment in both establishing a safe and therapeutic milieu but along side this the role of social, educational and therapeutic interaction with skilled staff has also been highlighted [60].

## Conclusion

Previous interviews with service users have highlighted both the role of both the environment and relationships in the patient experience [62,63]. Service users in this study identified the central role of relationships in the patient experience, and their accounts clarify the important elements of relationships and how they inform patient experiences. The user-led approach of this study impacts on all aspects of the research. It results in an understanding of service users' experiences of hospital which differs in content and emphasis from other previously undertaken academic and clinician led studies in this area. The implications of this research are twofold: while improvements in the environment in psychiatric hospitals are welcome, emphasis should be placed on developing practice to enhance the formation of therapeutic relationships with patients to have a positive impact on service users' experiences. Secondly, the value of user research lies not only in empowerment of people with mental illness but also in expanding the evidence base derived from psychiatric and health services research.

## Competing interests

The authors declare that they have no competing interests.

## Authors' contributions

HG contributed to the design of the study, collected and analysed the data and co-wrote this paper. DR contributed to the design of the study and analysis of the data and co-wrote this paper. MS was the Principal Investigator. He commented on this paper.

## Pre-publication history

The pre-publication history for this paper can be accessed here:


